# Mapping autoantibody targets of full-length C-reactive protein in systemic lupus erythematosus: importance for neutrophil function and classical complement activation

**DOI:** 10.3389/fimmu.2025.1578372

**Published:** 2025-05-15

**Authors:** Jesper Karlsson, Lina Wirestam, Hanna Duàn, Suhana Ahmad, Daniel Appelgren, Helena Enocsson, Jonas Wetterö, Christopher Sjöwall

**Affiliations:** ^1^ Division of Inflammation & Infection, Department of Biomedical and Clinical Sciences, Linköping University, Linköping, Sweden; ^2^ Division of Diagnostics and Specialist Medicine, Department of Health, Medicine, and Caring Sciences, Linköping University, Linköping, Sweden

**Keywords:** autoantibody, C-reactive protein, epitope, inflammation, lupus nephritis, neutrophils, pentraxins, systemic lupus erythematosus

## Abstract

C-reactive protein (CRP) is an important pattern recognition molecule of innate immunity. Autoantibodies targeting CRP are common in patients with systemic lupus erythematosus (SLE) and the levels correlate with disease activity. The purpose of this study was to investigate binding sites of IgG autoantibodies on the full linear sequence of CRP and identify potential associations with clinical variables in well-characterized SLE patients; a secondary aim was to investigate the effect of an epitope-based synthesized peptide motif on neutrophil functions. The levels of anti-CRP and SLE-associated antibodies were assessed, and a microarray-based linear epitope mapping was performed to detect binding sites on the full CRP monomer. We observed that anti-CRP antibodies bind to a variety of linear epitopes with a higher prevalence in SLE compared to healthy blood donors. Eleven unique epitopes were identified, of which five were found exclusively in SLE. Furthermore, we show that patients with anticardiolipin IgG and/or anti-β2GPI IgG antibodies have a higher number of positive CRP epitopes, and some CRP autoantibody-specificities associate with antiphospholipid antibodies, disease activity, and classical complement activation. In addition, one identified motif was selected, synthesized, and used for studying neutrophil function. This peptide showed modulatory capacity on neutrophil oxidative burst and chemotaxis, but not on neutrophil extracellular trap formation. Our results implicate a wide variation of anti-CRP autoantibody binding motifs of the linear structure of CRP in SLE patients. Some epitopes have the potential to modify innate host responses of relevance to the pathogenesis of SLE.

## 1 Introduction

The pentraxin proteins are phylogenetically highly conserved and involved in pattern recognition, opsonization and elimination (1). The family includes short and long pentraxins where C-reactive protein (CRP) is the most well-known short pentraxin, while pentraxin-3 (PTX3) serves as the prototypic long pentraxin. Pentraxins are considered ancestors of antibodies and share functions such as complement activation and Fcγ receptor interaction (2). CRP is produced in the liver, mainly as an outcome of interleukin (IL-) 6 stimulation. As a widely used biomarker in clinical routine, CRP levels can aid in differentiating between bacterial and viral infections, as well as being a reflector of systemic inflammation in many conditions (3).

Systemic lupus erythematosus (SLE) is an autoimmune disease characterized by a high number of different autoantibodies, clearance deficiency, and immune complex deposition (4). Several independent groups have reported autoreactivity to CRP and demonstrated that immunoglobulin (Ig) G anti-CRP antibodies can be found in more than a third of patients with SLE, usually with levels correlating with disease activity and associating with proliferative lupus nephritis (LN) (5–8). Still, anti-CRP antibodies have also been detected in other conditions (9–11). In SLE, circulating CRP levels do not reliably reflect ongoing inflammation as levels often remain relatively low despite increased inflammation and high IL-6 levels. Although CRP is indeed found in tissues deposited with immune complexes (12) as well as on the surfaces of extracellular vesicles (EVs) in SLE (13), this blunted response appears to mainly be a consequence of type-I interferon dysregulation as well as polymorphisms of the CRP gene *rs*1205 (14).

Native pentameric CRP (pCRP) consists of five identical 206 amino acid (a.a)/23kDa subunits and can reach vastly elevated levels within hours of an inflammatory onset (15). The known biological functions of CRP are connected to opsonization (directly or via classical complement activation), Fcγ-receptor binding and clearance of pathogens, dying endogenous cells and debris (16). Native pCRP can be irreversibly disassociated into monomeric CRP (mCRP). Like pCRP, mCRP possesses the ability to activate the classical complement pathway through C1q binding. Furthermore, both CRP isoforms can bind inhibitory factors such as factor H to modulate complement activation, thereby hindering the end-point membrane attack-complex of the classical complement pathway (17). A growing body of evidence highlights mCRP as the main CRP isoform to drive inflammation at local sites (18). In myocardial infarction for instance, dissociation of pCRP to mCRP on the surface of EVs, followed by EVs binding to endothelium and promoting inflammation has been observed (19). Furthermore, CRP accumulation in cardiovascular lesions has been indicated to aggravate inflammation by complement and immune cell activation (20–23). Recently, we showed that an increased presence of mCRP-bound EVs in plasma, as well as an altered mCRP/pCRP ratio in circulation, is associated with active SLE (13, 24).

Anti-CRP antibodies were first described in the 1980’s and have later been shown to target epitopes not accessible in the soluble native, pentameric structure of CRP (5). These “hidden” neoepitopes are exposed once the pentameric structure is compromised and pCRP has dissociated into mCRP. When pCRP deposits in an inflamed or damaged tissue it initially converts to pCRP*, an intermediate conformation state which is recognized by antibodies targeting neoepitopes while still retaining its pentameric integrity (25). Previously, Li et al. proposed a.a 35–47 as a major linear epitope of anti-CRP antibodies in patients with LN. Furthermore, they showed that this region of mCRP interacts with factor H *in vitro*, and that anti-mCRP a.a. 35–47 could inhibit mCRP binding to factor H (26). Additionally, Liu and colleagues found that combined measurement of anti-mCRP a.a 35–47 and anti-C1q linear epitope A08 can potentially predict a poor renal outcome (27).

In SLE, anti-CRP antibody levels are seen to vastly fluctuate over time, usually with higher levels during active disease (5). Furthermore, anti-double-stranded (ds) DNA and anticardiolipin (aCL) antibodies have been observed in higher prevalence of anti-CRP positive subjects (28); and anti-CRP antibodies may induce pro-inflammatory responses through macrophage clearance of anti-CRP opsonized apoptotic material (29). In LN, anti-mCRP antibodies have been suggested to have pathogenic potentials and are seen to associate with disease activity and prognosis (8, 12). Elucidating autoantibody target epitopes on CRP could increase understanding of the pathogenesis and potentially lead to the advancement of novel biomarkers.

The aim of this study was to investigate the full primary sequence of the CRP monomer for linear binding sites of IgG anti-CRP antibodies and identify potential associations with clinical parameters of patients with SLE. Prior studies have shown that peptides generated from CRP can alter the inflammatory neutrophil response (30–32). Hence, a subsequent secondary aim was to investigate potential neutrophil regulatory functions of an identified anti-CRP antibody epitope. To accomplish this, a complete screen for CRP autoantibody binding to linear epitopes was first executed, followed by functional studies on potential modulation of neutrophils for a selected sequence.

## 2 Materials and methods

### 2.1 Study populations

IgG antibody binding epitope mapping was conducted on sera from 42 patients diagnosed with SLE and 11 age- and sex-matched healthy blood donors (HBD) from the Clinical Immunology & Transfusion unit, University Hospital in Linköping. All patients took part of the prospectively enrolling regional quality register *Clinical Lupus Register in North-Eastern Gothia* (33), based at the University Hospital in Linköping. All patients fulfilled the 1982 American College of Rheumatology (ACR-82) (34) and/or the 2012 Systemic Lupus International Collaborating Clinics classification criteria (35). SLE disease activity index 2000 (SLEDAI-2K) was used to assess disease activity (36). Patients with SLEDAI-2K ≥5 were considered as having active SLE, as commonly defined (37). The SLICC/ACR damage index (SDI) was used to estimate irreversible organ damage (38). Characteristics of patients and HBD are detailed in **Table 1**.

**Table 1:** Detailed characteristics of the included patients and healthy blood donors.

	SLE (*n* = 42)	HBD (*n* = 11)
Background variables		
Age (mean (SD))	51 (16)	53 (7)
Female gender, *n* (%)	39 (93)	9 (82)
Ongoing anti-inflammatory agents		
Glucocorticoids, *n* (%)	28 (67)	
Hydroxychloroquine, *n* (%)	22 (52)	
Methotrexate, *n* (%)	7 (17)	
Azathioprine, *n* (%)	5 (12)	
Mycophenolate mofetil, *n* (%)	5 (12)	
Sirolimus, *n* (%)	2 (5)	
Rituximab, *n* (%)	1 (2)	
Disease variables		
Disease duration, years (mean (SD))	13 (11)	
SLEDAI-2K (median (IQR))	1 (0–4)	
SDI (median (IQR))	1 (0–3)	
Laboratory variables		
Hemoglobin, g/L (mean (SD))	132 (13)	
Leukocyte count, 10^9^/L (median (IQR))	6.3 (4.5–7.9)	
Erythrocyte sedimentation rate, mm/h (median (IQR))	13 (8–21)	
Plasma creatinine, µmol/L (median (IQR))	71 (63–82)	
Estimated GFR, ml/min/1,73m^2^ (median (IQR))	78 (66–94)	
C3, g/L (median (IQR))	1.0 (0.9–1.2)	
C4, g/L (median (IQR))	0.2 (0.1–0.2)	
CRP, mg/L (median (IQR))	2.4 (0.9–6.9)	
Anti-CRP antibodies, units (median (IQR))	17.5 (5.8–75.3)	
Anti-CRP antibody-positive, *n* (%)	26 (62)	
Clinical phenotypes (ACR-82 definitions)		
1. Malar rash, *n* (%)	20 (48)	
2. Discoid rash, *n* (%)	8 (19)	
3. Photosensitivity, *n* (%)	23 (55)	
4. Oral ulcers, *n* (%)	4 (10)	
5. Arthritis, *n* (%)	34 (81)	
6. Serositis, *n* (%)	14 (33)	
7. Renal disorder, *n* (%)	7 (17)	
8. Neurologic disorder, *n* (%)	2 (5)	
9. Hematologic disorder, *n* (%)	23 (55)	
10. Immunologic disorder, *n* (%)	19 (45)	
11. IF-ANA, *n* (%)	42 (100)	

CRP, C-reactive protein; GFR, glomerular filtration rate; HBD, healthy blood donors; IF-ANA, antinuclear antibodies analyzed by immunofluorescence technique; IQR, interquartile range; SD, standard deviation; SDI, Systemic Lupus International Collaborating Clinics/American College of Rheumatology damage index; SLE, systemic lupus erythematosus; SLEDAI-2K, SLE Disease Activity Index 2000.

All serum samples were stored in –80°C until analysis. For the neutrophil isolation experiments, whole blood from another 16 HBD (mean age (range): 50 (24–67) years, 18.8% female gender) was collected at GeBlod (Linköping, Sweden) at the time of blood donation.

### 2.2 Anti-CRP, antiphospholipid and antinuclear antibodies

Anti-CRP IgG antibodies were measured utilizing an in-house ELISA previously described (9). The results are expressed in arbitrary units (AU) as percentage of a positive reference sample obtained from a patient with SLE showing high anti-CRP levels. A positive result was determined using the 95^th^ percentile of 100 HBD as described (9).

Anti-β2GPI and aCL antibodies were assessed using Anti-Cardiolipin IgG/IgM and Anti-β2-Glycoprotein I IgG/IgM ELISA kits (ORGENTEC Diagnostika GmbH, Mainz, Germany) according to the manufacturer’s instructions and recommended cut-offs. aPL positivity was defined as being positive for either aCL and/or anti-β2GPI antibodies of either IgG and/or IgM isotype.

All samples were analyzed for IgG-ANA fine-specificities FIDIS™ Connective Profile, Solinium software version 1.7.1.0 (Theradiag, Croissy-Beaubourg, France) at the Clinical Immunology Laboratory, Linköping University Hospital (39). This addressable laser bead assay (ALBIA) simultaneously measures autoantibodies to Ro52/SSA, Ro60/SSA, La/SSB, Smith antigen (Sm), Smith antigen/ribonucleoprotein (Sm/RNP) complex, U1 RNP (U1-RNP), centromere B, Scleroderma-70, dsDNA, ribosomal P protein and histone.

### 2.3 Clinical routine laboratory measurements

The Clinical Chemistry unit at Linköping University Hospital, and the Rudbeck Laboratory, Department of Immunology, Genetics and Pathology, Uppsala University, carried out clinical routine analyses (e.g., blood cell counts, erythrocyte sedimentation rate (ESR), etc.), measured complement proteins (C3, C4, and C3d by nephelometry), and assessed classic hemolytic complement function (40). Plasma CRP and IL-6 levels were measured using high-sensitive turbidimetry (detection limit 0.15mg/L) and colorimetric immunoassay (detection limit 1.5ng/L), respectively.

### 2.4 Neutrophil isolation

Heparinized whole blood on Polymorphprep (Axis-Shield, Dundee, Scotland) and Lymphoprep (Serumwerk Bernburg AG, Bernburg, Germany) was centrifuged for 30 min at 480g in room temperature (RT). The neutrophil fraction was collected and followed by red blood cell lysis with ultrapure H_2_O. Modified Ringer’s phosphate buffer without Ca^2+^ (KRG^-Ca^; 120mM NaCL, 4.9mM KCl, 1.2mM MgSO_4_ • 7H2O, 1.7mM KH_2_PO_4_, 8.3mM Na_2_HPO_4_ • 2H_2_O, 10mM glucose; pH 7.3) was added and the cells were centrifuged for 10 min at 300g in 4°C. Erythrocyte lysis was performed twice with cold ultrapure H_2_O for 30s on ice followed by 300g centrifugation in 4°C and KRG^-Ca^ washing. Neutrophils were resuspended in modified Ringer’s phosphate buffer with 1mM Ca^2+^ (KRG) or RPMI 1640 (2mM glutamine, 2% FBS), counted, and kept on ice. All isolations were initiated within 2 hours of whole blood collection.

### 2.5 Linear epitope mapping

The full sequence of the CRP monomer was printed in 15 a.a sequences with 14 a.a overlap using PEPperCHIP Custom Peptide Microarray (PEPperPRINT^®^ GmbH, Heidelberg, Germany). The printed sequence was enclosed within a grid of influenza hemagglutinin (HA) antigen. PBS-Tween (PBST) was added for 15 min before blocking with blocking buffer (PBST, 1% human serum albumin) for 30 min, followed by 45 min incubation with goat anti-human IgG conjugated with Dylight 680 (Thermo Fisher Scientific, Waltham, MA, USA). The arrays were submerged in Tris-HCL, pH 7.4, and air-dried before scanning for background fluorescence levels. After scanning, the arrays were blocked once more in blocking buffer for 15 min followed by addition of serum samples (1:1000 dilution in PBST) and overnight incubation in 4°C. Once more, anti-human IgG was added, and the arrays were submerged in Tris-HCL as described above followed by scanning to ensure adequate signal was achieved. Arrays were blocked for 15 min followed by 45 min incubation with the addition of anti-HA antibody conjugated with Dylight 680. A final submersion in Tris-HCL was performed before final scanning of the arrays. Washing was performed between steps using PBST. All incubations were done in room temperature on an orbital shaker unless otherwise described. Odyssey CLx Imaging system (LI-COR Biosciences, Lincoln, NE, USA) were used for array scanning. Images were obtained with Image Studio software version 1.0.11 at 21 µm resolution and analyzed using PepSlide Analyzer software version 2.2.8 (Sicasys Software GmbH, Heidelberg, Germany). Cut-off level for positivity was set to 2× the background fluorescence. Epitopes were determined as the minimum number of a.a in overlapping positive regions with at least three amino acids overlapping.

### 2.6 In-house peptide ELISA

Selected peptides based on linear epitope mapping results were obtained using Fmoc solid phase based custom peptide synthesis service (Thermo Fisher Scientific) with >95% purity. The peptides were dissolved in dimethyl sulfoxide (DMSO), aliquoted and stored below -70°C before usage. As peptide control, a scrambled version containing the same amino acids and similar charge was designed (**Supplementary Material 1**). To assess autoantibody binding of the peptides, an in-house ELISA was developed. High binding 96-well half-area polystyrene plates (Corning, NY, USA) were coated with 1µM synthetic peptide in bicarbonate buffer (pH 9.6) and incubated overnight at 4°C. Non-coated wells were incubated in bicarbonate buffer. The plates were blocked with blocking buffer (10mg/ml human serum albumin; Albunorm^®^, Octapharma, Stockholm, Sweden; in PBS-Tween 0.05%) for 2 hours at RT. After 1 hour incubation at RT with human serum (diluted 1:50 in PBS-Tween), alkaline phosphatase-conjugated polyclonal rabbit anti-human IgG (diluted 1:2000 in PBS-Tween; Abcam, Cambridge, UK) was added and the plates were incubated for 1 hour in RT. para-Nitrophenylphosphate was added and optical density (OD) was measured after 15 min at 405nm with 630nm as reference (Sunrise plate reader, Tecan, Männedorf, Switzerland; Magellan ver. 7.1 software, Tecan).

### 2.7 Neutrophil oxidative burst

Reactive oxygen species (ROS) were measured using luminol-amplified chemiluminescence. Neutrophils (10^6^ cells/ml) in KRG were added to black 96-well plates (Nunc™, Thermo Fisher Scientific) with luminol (50µM) and horseradish peroxidase (4U/mL) followed by pre-heating of the plates in 37°C for 5 min. Phorbol myristate acetate (PMA; 10^-7^M) addition stimulated the neutrophils and the total NADPH oxidase activity was measured with a luminometer (Hidex Chameleon II, Hidex, Turku, Finland). Measurements were done every 30 seconds for 15 min in 37°C in reversed series duplicates. During the 5 min of pre-heating, incubations were done with 10^-9^, 10^-8^,10^-7^M synthesized peptides or DMSO corresponding to the highest concentration (not exceeding 0.01%). Values are calculated as percentage of negative control and presented as area under curve. Non-stimulated cell suspension was used as negative control.

### 2.8 Three-dimensional chemotaxis

Neutrophil 3D chemotaxis was examined in collagen gel (2mg/ml Bovine Collagen I, **Supplementary Table S1**) using µ-Slide Chemotaxis assay (ibiTreat coated surface; ibidi GmbH, Gräfelfing, Germany) according to the instructions of the manufacturer. Isolated cells resuspended in RPMI were put in the middle chamber with collagen gel and incubated for 30 min in 37°C, 5% CO_2_. Peptide-incubations were performed at this stage. Cell morphology was assessed during and after gelation using an inverted light microscope. Formylmethionine-leucyl-phenylalanine (fMLF) (10^-8^M) was used as chemoattractant unless stated otherwise. Time-lapse images of all three chambers were captured sequentially every minute for 60 min using digital image analysis (Nikon spinning disc; Nikon Instruments, Amstelveen, Netherlands; NIS-Elements AR Imaging Software, Nikon) at 10x magnification, numerical aperture 0.5. The cells were tracked frame by frame using the “Manual Tracking” plugin of the Fiji software (41).

### 2.9 NET remnant assay

Neutrophils (50–000 cells/well) were added to black 96-well optical bottom plates (Thermo Fisher Scientific) and peptides or the NADPH oxidase inhibitor diphenyleneiodonium chloride (DPI) were added followed by 5 min incubation in 37°C. PMA (20nM) was added, and the plates were incubated for 4h in 37°C. Sytox Green (2.5µM), a cell impermeable DNA binding dye, was added to each well followed by 15 min incubation in 37°C. Fluorescence was measured at 475nm_EX_, 500-550nm_EM_. Furthermore, NET remnants (hereon referred to as NETs) were isolated by the addition of DNAse (10U/ml) and detected using an ELISA recognizing DNA and myeloperoxidase (MPO) complexes as previously described (42). In short, 96-well MaxiSorp plates (Thermo Fisher Scientific) were coated overnight at 4°C with polyclonal anti-MPO (Agilent DAKO, Santa Clara, CA, USA) before the addition of samples, standards (standard prepared as previously described (42)), and controls. These additions were diluted with anti-DNA antibodies labelled with peroxidase (detection antibody of Human Cell Death Detection ELISAPLUS kit; Roche diagnostics GmbH, Mannheim, Germany) and incubated before substrate (ABTS; Roche Diagnostics GmbH) was added and the plate was read at 405nm using a VersaMax ELISA microplate reader (Molecular Devices, Sunnydale, CA, USA).

### 2.10 Statistical analysis

The data was tested for normality using Shapiro-Wilk tests and outliers were examined. For non-parametric data, Mann-Whitney *U* tests were utilized between non-paired samples, and Spearman’s rank correlation coefficient test for correlation analysis. Comparisons between binary data were done using exact χ^2^-test. For non-parametric data between multiple paired samples, Friedman tests were used followed by Dunn’s multiple comparisons test. Wilcoxon signed-rank test was used for non-parametric data between two groups of paired samples. Data was analyzed using SPSS statistics 29 (IBM Corp., Armonk, NY, USA) or GraphPad Prism 10 (GraphPad Software, Boston, MA, USA). A p-value of <0.05 was considered statistically significant.

### 2.11 Ethical considerations

Written and oral informed consent was provided from all subjects included. The study protocol was approved by the Regional Ethics Review Board in Linköping (decision no. M75-08) and Swedish Ethical Review Authority (decision no. 2023-03937-01) and was performed according to the Declaration of Helsinki.

## 3 Results

### 3.1 Anti-CRP antibodies and linear epitope positivity in SLE and HBD

**Figure 1:**
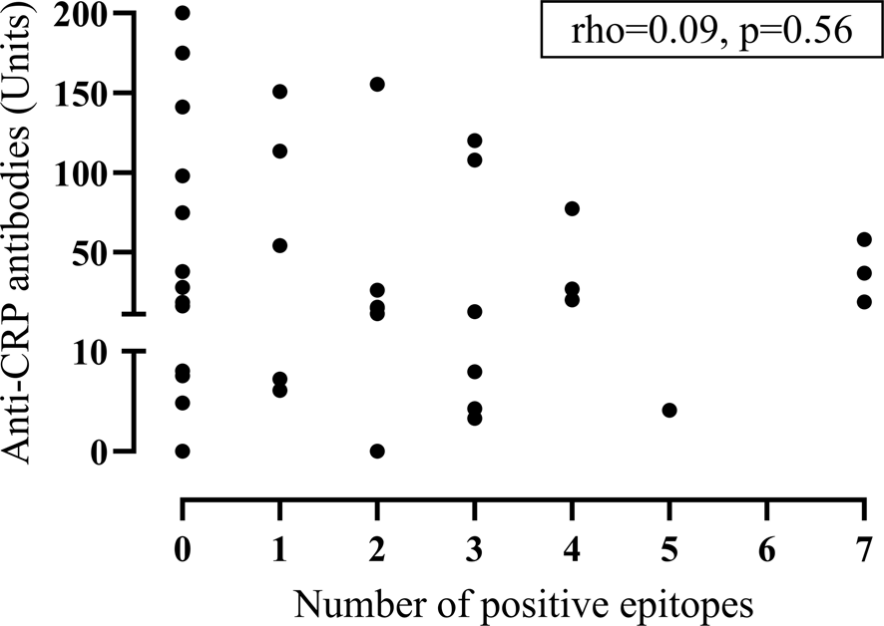
Dot-plot between anti-CRP antibodies and epitope positivity. The graph displays anti-CRP antibodies versus the number of positive epitopes obtained with microarray-based linear epitope mapping in patients with systemic lupus erythematosus (*n*=42). (CRP, C-reactive protein).

Patients with serum samples containing anti-CRP antibodies showed a higher ESR (median(interquartile range, IQR): 14.5(9.5-23.8) vs. 10(7-13)mm/h, p<0.05), higher levels of C3d (median(IQR): 3.8(3.1–4.3) vs. 2.5(1.5–3.7)g/L, p<0.05), a decreased function of the classical complement pathway (median(IQR): 96(71–107) vs. 107(81–118)%, p<0.05) as well as higher levels of both aCL (median(IQR): 1.71(0.48–6.75) vs. 0.28(0.28–1.1)U/ml, p<0.001) and anti-β2GPI IgG antibodies (median(IQR): 0.98(0.09–7.35) vs. 0.02(0.02–0.36)U/ml; p<0.01) compared to anti-CRP negative patients. Being positive for anti-CRP was not significantly associated with having aPL antibodies (p=0.27) and anti-CRP levels did not correlate significantly with the number of positive epitopes in the CRP primary sequence (rho=0.09, p=0.564; **Figure 1**).

**Figure 2:**
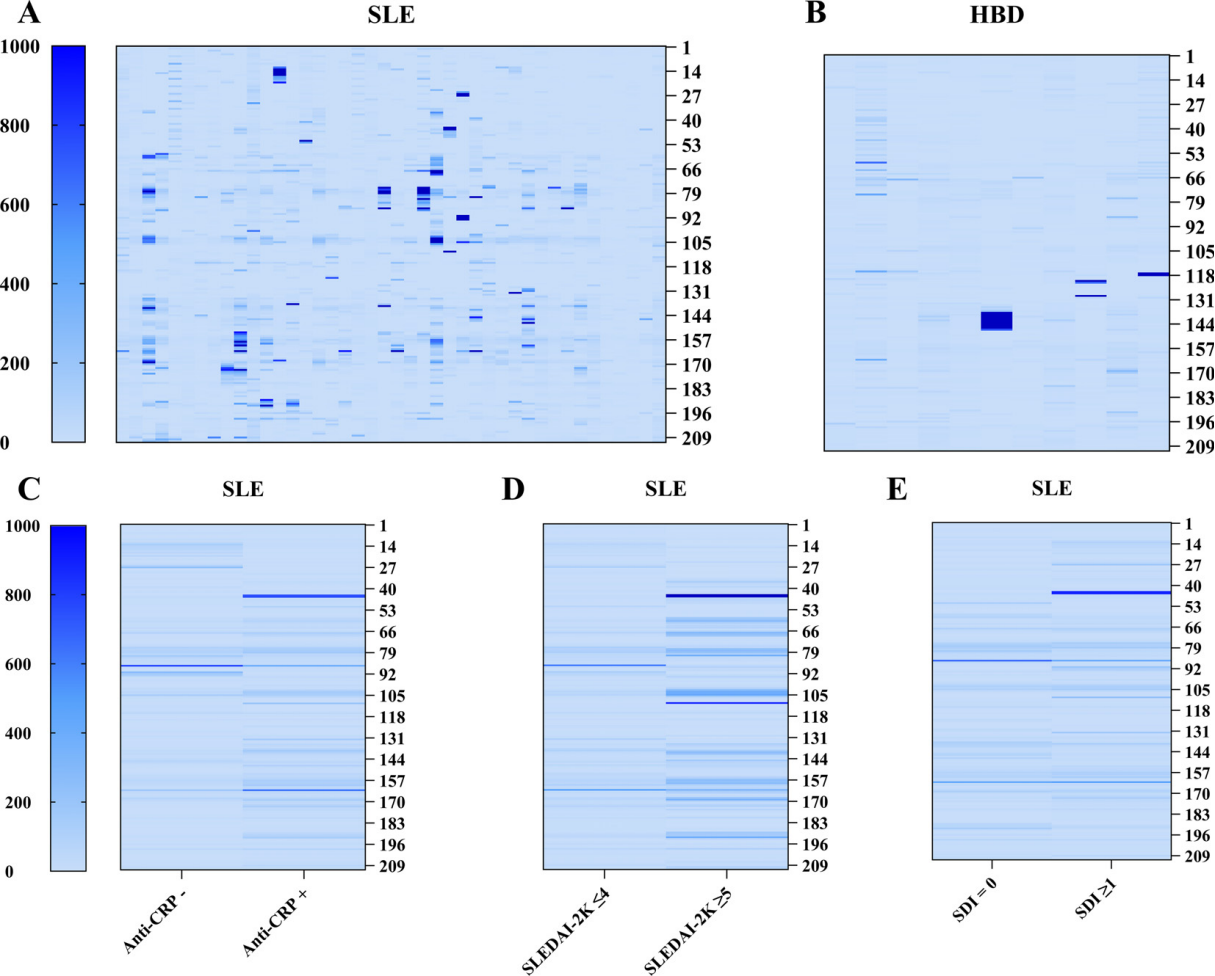
Heatmap representation of autoreactivity against CRP obtained with microarray-based linear epitope mapping of the full CRP monomer. **(A, B)** Individual signal intensity of IgG autoantibody reactivity against full-length CRP for subjects with SLE (*n*=42) and HBD (*n*=11). **(C–E)** Mean signal intensity of IgG autoantibody reactivity against motifs of full-length CRP comparing anti-CRP negative (anti-CRP–; *n*=16) vs. anti-CRP positive (anti-CRP+; *n*=26) patients with SLE, patients with (*n*=6) and without (*n*=36) active disease, and no damage (*n*=20) vs. irreversible organ damage (any organ system; *n*=22). Each column represents one subject **(A, B)** or the mean value of a group **(C–E)**. Each row represents a 15 amino acid long sequence covering the full length of the protein with 14 amino acids overlap and 7 amino acid GS repeats elongated before and after the protein sequence. (CRP, C-reactive protein; HBD, healthy blood donors; SDI, Systemic Lupus International Collaborating Clinics/American College of Rheumatology damage index; SLE, systemic lupus erythematosus; SLEDAI-2K, SLE Disease Activity Index 2000).

In total, eleven epitopes of CRP were identified, where six of these were found in both patients and HBD. Patients with SLE were more frequently positive for CRP epitopes compared to HBD with 24 patients (57%) and 4 HBD (36%) being positive for ≥1 epitope (**Figures 2A, B**). The median number of positive epitopes was higher in SLE compared to HBD (median(range): 1(0–7) and 0(0–4) respectively). Among patients positive for any linear epitope, 67% were anti-CRP positive, while patients without any positive CRP linear epitopes were anti-CRP positive in 56% of the cases. No significant differences were found between anti-CRP positive and negative patients with regards to the number of positive linear epitopes (median: 1.5 vs. 0.5) or the signal intensity of positive epitopes (median: 892 vs. 652 AU; **Figure 2C**). CRP epitope-positive patients had a longer disease duration (median(IQR): 15(6–23) vs. 8(7–12) years, p<0.05), lower levels of IL-6 (median(IQR): 1.9(0.9–6.7) vs. 8.4(3–24.5)pg/ml, p<0.05) and decreased function of the classical complement pathway (median(IQR): 94(74–107) vs. 105(90–121)%, p<0.05). No significant differences were found in levels of CRP, anti-CRP antibodies, disease activity assessed by SLEDAI-2K, SDI, or ANA levels (anti-dsDNA, anti-ribosomal P protein, anti-SSA/Ro60, anti-SSA/Ro52, anti-SSB/La, anti-SmRNP) between patients positive or negative for CRP epitopes.

### 3.2 Linear epitopes, antiphospholipid antibodies and disease activity

Positivity against the epitope PDE^168–170^ was most common for patients (*n*=13), followed by DIGN^155-158^ (*n*=12), SFGGNFEGSQSL^141-152^ (*n*=10), and SEIL^80-83^ (*n*=10). All these epitopes were positive in at least one control. Positivity against PDE^168-170^ (*n*=2 HBD), VRKSLKK^117-123^ (*n*=2 HBD, *n*=1 SLE), and ILGQ^134-137^ (*n*=1 HBD, *n*=8 SLE) were also found among HBD. CLH^36-38^ (*n*=2), EILIF^62-66^ (*n*=2), SWE^99-101^ (*n*=7), YEVQG^192-196^ (*n*=5), and QLWP^203-206^ (*n*=3) were positive exclusively in patients. Two epitopes were significantly more prominent in active compared to inactive SLE: SEIL^80-83^ (p<0.05), and DIGN^155-158^ (p<0.05). Furthermore, SEIL^80–83^ was associated with a decreased function of the classical complement pathway (median(IQR): 78(63–95) vs. 104(88–119)%, p<0.01) and aPL positivity (p<0.05).

Patients with active SLE associated with being positive for any CRP epitope (p<0.05) and displayed a higher number of positive epitopes, but not higher intensity value of positive epitopes, compared to non-active disease (median(IQR): 4(1.8–7) vs. 0.5(0–2.8) epitopes, p<0.01 and 976(678–1117) vs. 737(516–1624) AU, p=0.54 respectively; **Figure 2D**). Furthermore, aPL IgG antibody-positivity (aCL and/or anti-β2GPI, *n*=5) was also associated with a higher number of positive CRP epitopes (median(IQR): 4(2.5–5.5) vs. 1(0–2.5) positive epitopes, p<0.01), but neither aPL positive (*n*=10) nor patients with active disease differed in CRP and anti-CRP antibody levels compared to aPL negative and non-active disease respectively. Additionally, in aPL positive patients, there was a strong correlation between the signal intensity of CRP epitopes and anti-CRP antibodies (rho=0.64, p<0.05) as well as between the signal intensity of CRP epitopes and SLEDAI-2K (rho=0.73, p<0.05) which was not observed in the aPL negative group. There was no significant correlation between anti-CRP levels and SLEDAI-2K in the aPL positive group (rho=0.46, p=0.18), and no significant difference was seen between aPL positive and negative patients regarding disease activity. The number and the signal intensity of positive epitopes did not differ significantly between patients with and without irreversible organ damage (median: 0.5 vs. 2, p=0.35 and 600 vs. 909, p=0.17 respectively; **Figure 2E**).

### 3.3 CRP^72–86^ peptide recognition and effects on neutrophil function

Based on the observed implication of the SEIL^80–83^ epitope in SLE, and previously described functions of that specific region of CRP (see Discussion), we decided to investigate if a slightly elongated, synthesized version of that epitope (CRP^72-86^) would have any biological effects on neutrophils *in vitro*.

**Figure 3:**
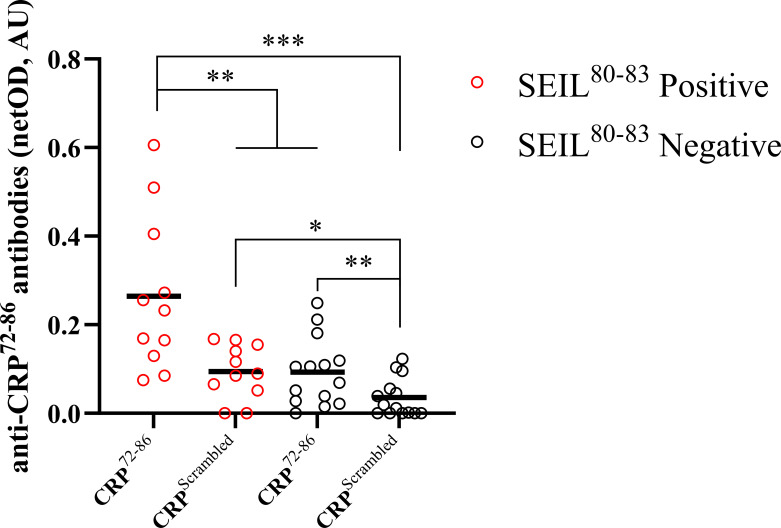
Comparison between recognition of synthesized peptides using an in-house ELISA. The graph shows autoantibody recognition of two CRP-based synthesized peptides for subjects positive (*n*=11) and negative (*n*=14) for the SEIL^80–83^ epitope of CRP (assessed by linear epitope mapping). Groups include both SLE and HBD. The SEIL^80–83^ negative group did not have any positive CRP-epitopes assessed by linear epitope mapping. Results are presented as netOD by calculating OD values subtracted by background OD values obtained with non-coated surfaces and presented as median with individual values in AU. Negative netOD values were set to 0. Wilcoxon signed-rank tests were applied between paired samples and Mann-Whitney *U* tests were applied between non-paired samples. (AU, arbitrary units; CRP, C-reactive protein; ELISA, enzyme-linked immunosorbent assay; HBD, healthy blood donors; IQR, interquartile range; OD, optical density; SLE, systemic lupus erythematosus; *p<0.05; **p<0.01; ***p<0.001).

In-house peptide ELISA showed that CRP^72–86^ was recognized to a higher extent by SEIL^80–83^ positive than SEIL^80–83^ negative individuals. This was also true for the scrambled peptide, while a significantly higher signal was seen with CRP^72–86^ compared to the scrambled peptide in the SEIL^80–83^ positive group (**Figure 3**).

**Figure 4:**
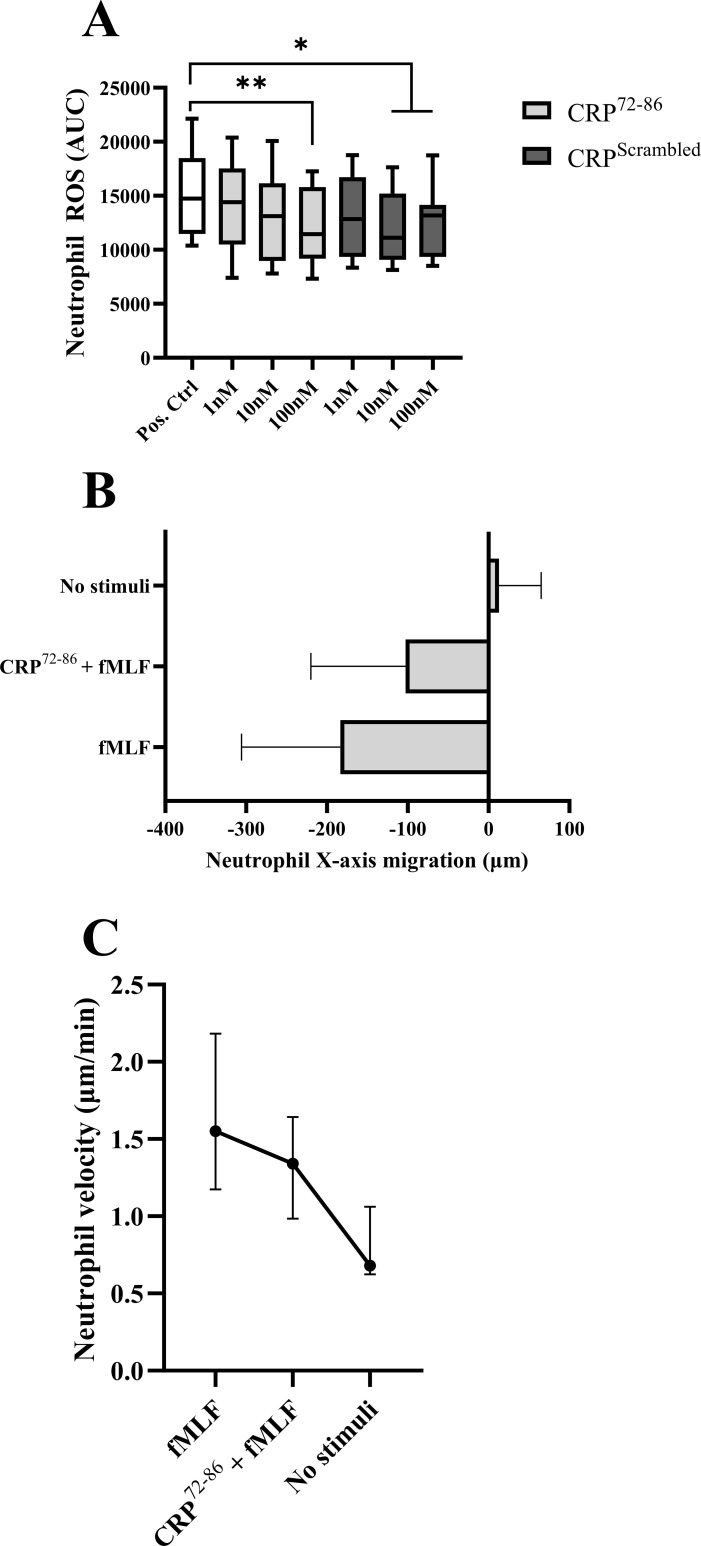
The effect of synthesized peptides on neutrophils isolated from HBD. **(A)** Paired samples of PMA-stimulated neutrophil oxidative burst with and without pre-incubation with synthesized peptides of different concentrations (*n*=9) using luminol-amplified chemiluminescence. Box and whiskers display median, IQR and range. Pos. Ctrl: PMA-stimulation with vehicle pre-incubation. Values were calculated as percentage of negative control (negative control standard deviation: ± 2.8%). **(B, C)** Neutrophil directional X-axis movement (origin was set to 0 for each cell) and velocity in a 3D-matrix with and without chemotactic stimulus (fMLF) and pre-incubation with a synthesized peptide (*n*=3; 15 cells were randomly selected and tracked manually for 60 min in intervals of one minute for each subject). Images were obtained using digital image analysis. The results are displayed as mean with SD **(B)** and median with range **(C)**. (AUC, area under curve; CRP, C-reactive protein; fMLF, formylmethionine-leucyl-phenylalanine; HBD, healthy blood donors; IQR, interquartile range; PMA, phorbol myristate acetate; *p<0.05; **p<0.01).

Neutrophils pre-treated with CRP^72–86^ displayed a dose-dependent inhibition of the PMA-triggered ROS production compared to vehicle control (**Figure 4A**), which was not seen for the scrambled peptide. A significant decrease was seen for pre-incubation with 100nM CRP^72-86^ (p<0.01), as well as 10nM and 100nM of the scrambled peptide (p<0.05). Direct neutrophil stimulation with CRP^72–86^ at 100nM showed no significant difference in neutrophil ROS production compared to basal ROS levels (not shown).

Chemotaxis stimulation with fMLF (10nM) revealed a decreased neutrophil directional movement when pre-treated with CRP^72-86^ (**Figure 4B**). The velocity of the cells also decreased while pre-treated with the CRP peptide (**Figure 4C**). Neither CRP^72–86^ nor DMSO had any stimulatory effect on directional movement compared to negative control (not shown).

Extracellular DNA/NET release by PMA (20nM) stimulation was not affected by CRP^72–86^ pre-treatment at 100nM as measured by Sytox Green fluorescence. No significant difference was found for NET levels from PMA-stimulated neutrophils with and without pre-treating with 100nM CRP^72–86^ peptide measured with MPO-DNA ELISA. Pre-incubating with the control peptide did not significantly alter PMA-stimulated Extracellular DNA/NET release measured by Sytox Green fluorescence or ELISA (**Supplementary Figure S1**).

## 4 Discussion

Autoantibodies in SLE have previously been shown to target a variety of constituents, also beyond nuclear constituents, probably contributing to the complexity of the disease pathogenesis and diverse clinical presentations (43). This study demonstrates that autoantigen motifs of CRP are scattered across many parts of the full amino acid sequence, with linear epitope mapping of SLE autoantibodies indicating a high coverage of the primary structure. Epitope-specific antibodies are furthermore associated with aPL antibodies, SLE disease activity, and complement activation.

Interestingly, healthy individuals were also positive against several of the identified epitopes. This is not unexpected since autoantibodies are known to occur in healthy individuals (44, 45). It may also be due to methodological aspects, such as the sensitive full-length screening approach of the microarray assay. Still, it’s also possible that autoreactivity to these epitopes may not necessarily be linked to pathology.

However, five CRP epitopes were exclusively found among the patients. At least three of these are positioned in regions of the primary sequence previously described for its intrinsic function in CRP, as a separate peptide, or as a target for anti-CRP antibodies. CRP^35–47^ is identified as a major epitope of mCRP in LN (8). In our cohort, only two patients were positive for this specific region, while both fulfilled the renal disorder criterion (ACR7), none of them had active nephritis (ongoing renal activity).

Several of the linear epitopes found in this study are located within previously described functional regions of CRP (26, 32, 46–53). CLH^36–38^ is responsible for the specificity of the cholesterol binding sequence (a.a 37-47), which binds to cholesterol, fibrinogen, collagen, fibronectin, C1q, and lipoprotein component ApoB after dissociation to mCRP (51, 53). Furthermore, CRP^35–47^ has been found to bind factor H, and antibodies targeting this region can inhibit the enhanced cofactor ability factor H achieves when bound to CRP (26), and anti-mCRP^35–47^ antibodies together with anti-C1qA08 antibodies are shown to associate with a worse renal prognosis in LN (27). Interestingly, the two epitopes most frequently found among the patients in this study were PDE^168–170^ and DIGN^155-158^, where both the asparagine of DIGN^155–158^ and the aspartic acid of PDE^168–170^ are involved in CRP binding to C1q (48, 50). The phenylalanine of EILIF^62–66^ has been shown to be involved in phosphocholine binding (49), and VRKSLKK^117–123^ is part of a small synthetic peptide shown to facilitate nuclear localization by microinjecting peptide-coupled proteins into cells *in vitro* (46). ILGQ^134–137^ together with SFGGNFEGSQSL^141–152^ spans the calcium-binding site of CRP which is closely localized to the phosphocholine binding site in its tertiary structure (47, 52). The epitope QLWP^203–206^ constitutes the last a.a of the primary sequence of CRP and is part of a peptide (CRP^201-206^) which is suggested to inhibit superoxide generation and decrease cytosolic calcium raise in stimulated neutrophils (superoxide generation stimulated with fMLF, PMA, calcium ionophore and the nonsteroidal anti-inflammatory drug benoxaprofen, while calcium raise was triggered by fMLF) (32). Furthermore, a recent study shows that anti-CRP^199–209^ antibodies in plasma of patients with LN associated with renal tubulointerstitial lesions and that immunization with CRP^199–209^ exacerbated tubulointerstitial lesions in mice (54). Two of the epitopes found herein (SWE^99-101^, YEVQG^192-196^) have to our knowledge not previously been described as anti-CRP antibody targets, nor with any known inherent functions of the sequences.

It was previously reported that anti-CRP positive patients with SLE display higher frequency of aPL (aCL antibodies of IgG and/or IgM and/or a positive lupus anticoagulant test) compared to anti-CRP negative subjects (28). In our study, anti-CRP positivity was not associated with aPL positivity, but the levels of aCL IgG and anti- β2GPI IgG were higher in anti-CRP positive individuals. Also, patients positive for aPL IgG antibodies displayed a higher number of positive CRP epitopes. Although this could be an effect caused by cross-reactivity, it is unlikely that this comprises the full effect. Furthermore, a previous study by Figueredo et al. showed no cross-reactivity between anti-CRP IgG and aCL IgG by testing the reactivity to CRP and cardiolipin-β2GPI substrates in patients with SLE positive for both anti-CRP and aCL after serum absorption of the two substrates (28). Lower levels of IL-6 and a decreased function of the classical complement pathway were also found in CRP epitope-positive patients. Potentially indicating a protective role of anti-CRP antibodies where the proinflammatory functions of CRP, mCRP specifically, is down-regulated while immune complex-mediated complement consumption progresses. However, since the positivity variable is binary, and no differences were seen for either CRP levels, anti-CRP antibody levels or disease activity between these patients, limited conclusions can be drawn from this. Patients with active disease had a higher number of positive CRP epitopes and associated with being positive for CRP epitopes, which connect well with previous findings of anti-CRP antibody levels correlating with disease activity (5). The lack of concordance between circulating anti-CRP antibody levels measured using the in-house ELISA and the findings of the linear epitope mapping is not surprising, as there are structural differences affecting recognition. Furthermore, the peptide immobilization strategy of the commercial microarray differs from the spontaneous adsorption of peptides in the ELISA coating. There was also a group of anti-CRP antibody-positive patients who did not achieve positivity for any CRP epitope. Although the cut-off for positivity differs between the two methods and is applied differently, this difference is probably partly due to discontinuous epitopes not recognized in the linear epitope mapping. Even though discontinuous epitopes are considered the most common type of B cell epitopes (55, 56), linear epitopes are still of interest, and analyzing autoantibodies targeting continuous linear epitopes can yield important information about the impact of different autoantibodies in health and disease. Since it is previously shown that CRP can be degraded by neutrophil elastase (57), it is also possible that some autoantibodies target degraded fragments that are not detected in an immunoassay utilizing native CRP. Another difference between the methods is that posttranslational modifications are not included in the microarray. With the full molecule CRP-ELISA-autoantibody detection approach it is furthermore not possible to know the exact orientation and/or the tertiary structure of the spontaneously adsorbed mCRP/pCRP at the plastic solid surface - liquid interphase. It is a possibility that some biologically potentially relevant epitopes of spontaneously adsorbed CRP are not readily available to autoantibody interaction in the ELISA.

SEIL^80–83^ was chosen to be synthesized for mechanistic studies because of its association with disease activity and complement function as well as its frequent positivity in the linear epitope mapping. The epitope is also contained within a region previously shown to inhibit superoxide generation and migration in neutrophils (32), and the glutamic acid part of SEIL^80–83^ is known to be vital for phosphocholine binding together with Phe (66) part of the above mentioned EILIF^62–66^ epitope (49). This made it an appealing epitope to synthesize and study potential biological effects on neutrophils. Interestingly, partial amino acid identity between CRP^77–90^ and heat-shock protein 60 (Hsp60) has been found and it has been shown that Hsp60 can be targeted by both polyclonal and monoclonal anti-CRP antibodies (58). This cross-reactivity emphasizes the difficulty of studying autoantibody epitopes in SLE due to the vast amount of autoantigen motifs.

Performing autoantibody-ELISA with short peptides as motifs is a major challenge, however the synthesized peptide CRP^72–86^ was recognized primarily by patients positive for the SEIL^80–83^ epitope, but also by SEIL^80–83^ negative patients and the scrambled sequence to some extent. The effects are small, and the netOD was low due to relatively high background values. However, the significantly stronger recognition of the peptide for SEIL^80–83^ epitope positive patients implicates that the synthesized peptide is a proper mimic of the epitope, and that it can be targeted by anti-SEIL^80–83^ autoantibodies.

Neutrophils have a crucial role in autoimmune diseases, such as SLE (59). The functional assays demonstrated that CRP^72–86^ could inhibit ROS production and decrease neutrophil directional movement, suggesting a modulatory effect on neutrophil activity. Pre-treatment with the scrambled-sequence control peptide led to a smaller, but still significant decrease of PMA-induced neutrophil ROS production. This is not surprising as the control peptide consists of the same amino acids, designed to have similar polarity across the sequence. Still, the lack of a relevant, but true, negative control without apparent effect remains a limitation. Pre-treatment with any of the peptides did not lead to visibly more static cells or cells with altered morphology, indicating that the viability was not affected. No difference was seen regarding NET release with peptide pre-treatment compared to controls, indicating that CRP^72–86^ might exert a more fine-tuned immune-modulatory effect without affecting proinflammatory necrosis/NETosis. The results from the functional studies herein are consistent with previous studies showing that CRP peptides can modulate immune cell functions (30–32). Recently, Karasu et al. reported that mCRP, but not pCRP, promotes NET formation as well as enhancing chemotaxis and ROS production in neutrophils (60). This is interesting since the peptide effects shown herein have an opposite effect on ROS production and no apparent effect on NET formation. Hypothetically, mCRP could induce the recruitment of phagocytic cells and further enhance clearance of debris, while CRP peptides, potentially degraded by neutrophil elastase, might have the opposite effect, thus operating as a negative feedback loop. The association between specific epitopes of anti-CRP antibodies and neutrophil functions underscores the potential role of these antibodies in immune modulation of relevance to SLE pathogenesis. Targeting or using specific CRP epitopes could be a therapeutic strategy in SLE, although further research is needed to elucidate the relationship of such epitopes and disease progression.

The included patients were exposed to different combinations of immunosuppressive and/or immunomodulatory agents, and we cannot exclude that this could have influenced the results. This is recognized as one of the limitations of this study.

To conclude, our study provides novel insights into anti-CRP antibodies in SLE, highlighting their broad coverage of the primary sequence of the protein, and indicating that the clinical associations differ among different anti-CRP antibodies. These antibodies, and anti-SEIL^80–83^ antibodies specifically, associated with disease activity, complement function, and aPL antibodies. Furthermore, small peptides generated from CRP might provoke modulatory effects on neutrophil function, exhibiting autoantibody effects against different epitopes. The results in this study are limited to linear epitopes, and future research should investigate the role of specific anti-CRP autoantibodies with different antigen reactivities (including discontinuous epitopes) in lupus pathogenesis and in relation to different organ manifestations.

## Data Availability

The original contributions presented in the study are included in the article/**Supplementary Material**. Further inquiries can be directed to the corresponding author.
