# Supplementary material for: Mapping autoantibody targets of full-length C-reactive protein in systemic lupus erythematosus: importance for neutrophil function and classical complement activation
Source: Front Immunol. 2025 May 15;16:1578372. doi: 10.3389/fimmu.2025.1578372 (PMC12119686; doi:10.3389/fimmu.2025.1578372)
Supplement: Supplementary file 1 [file SupplementaryFile1.docx]

Supplementary Material

Supplementary Material 1

## Peptide design

A scrambled peptide was generated from the peptide sequence GYSFTVGGSEILFEV. A random scrambled sequence was generated using “Tool for Scrambling Protein or Peptide Sequences” from peptidenexus.com (1). From the generated scrambled peptide, amino acids were re-positioned manually according to a scoring system developed for the “Wise Shuffling” application (2). For example, original sequence motifs, certain motifs, same amino acid repetition and reverse order neighbors were avoided. The charge pattern of the original peptide was conserved to the highest possible extent. The final peptide sequence was as follows: LFTISEGYEVGVSGF.

1. Peptidenexus.com, Tool for Scrambling Protein or Peptide Sequences. https://peptidenexus.com/article/sequence-scrambler, 2024 (accessed 23 December 2024).

2. Farkas, Ö., E. Lajko., M. Kalabay, K.T. Enyedi. How to Scramble*? A “Wise Way” to Achieve a Useful Control Peptide Sequence*. SSRN, 2022. Available at SSRN: https://ssrn.com/abstract=4363258 or <http://dx.doi.org/10.2139/ssrn.4363258>
