# Supplementary material for: Mapping autoantibody targets of full-length C-reactive protein in systemic lupus erythematosus: importance for neutrophil function and classical complement activation
Source: Front Immunol. 2025 May 15;16:1578372. doi: 10.3389/fimmu.2025.1578372 (PMC12119686; doi:10.3389/fimmu.2025.1578372)
Supplement: Supplementary file 2 [file SupplementaryFile2.docx]

Supplementary Material

Supplementary Figure S1


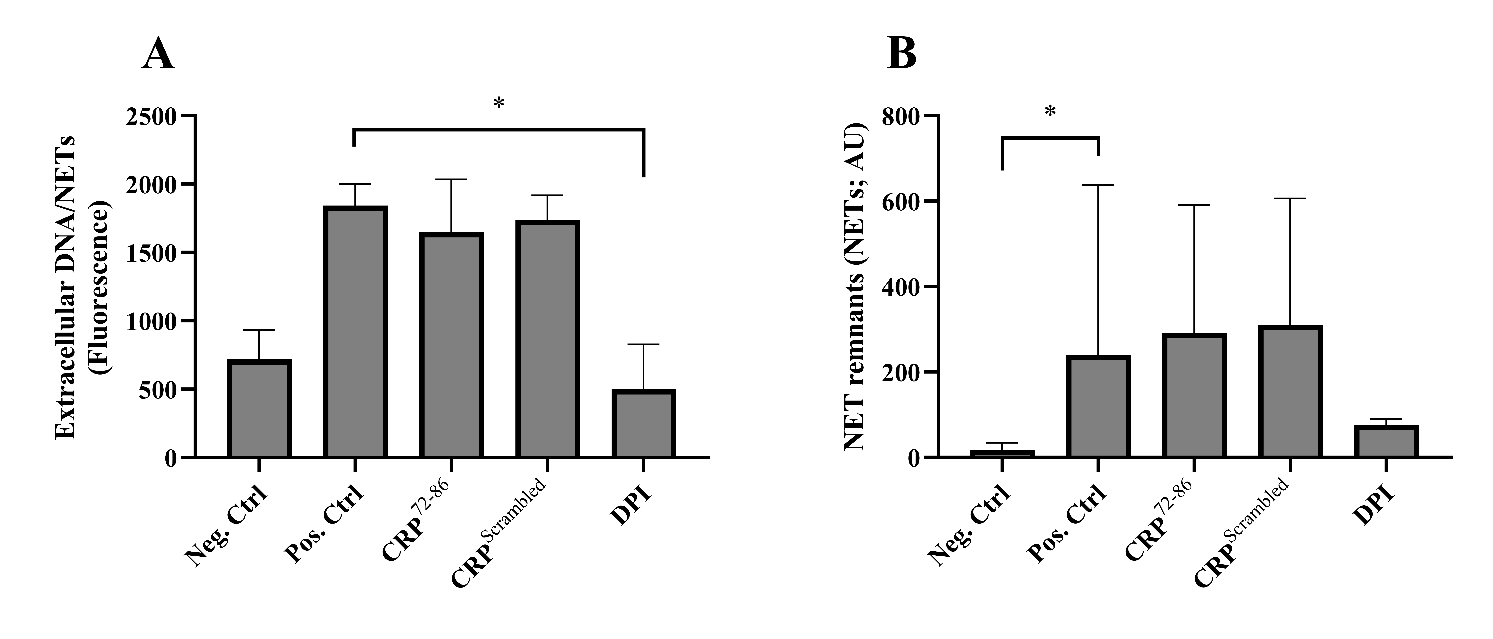


**Supplementary Figure 1.** The effect of synthesized peptides on the stimulated release of neutrophil extracellular DNA/NETs of neutrophils isolated from HBD. (A) Comparisons of extracellular DNA/NETs released by PMA-stimulated (20nM) neutrophils with and without pre-incubation with synthesized peptides or the NADPH oxidase inhibitor DPI using fluorescence (Sytox Green DNA binding cell impermeable dye). (B) Comparisons of NET remnants/NETs released by PMA-stimulated (20nM) neutrophils with and without pre-incubation with synthesized peptides or DPI using MPO-DNA ELISA. Pos. Ctrl: PMA-stimulated neutrophils pre-incubated with vehicle. Neg. Ctrl: Non-stimulated neutrophils pre-incubated with vehicle. *n*=5 paired samples in A and B. Bars display median with IQR. (AU, arbitrary units; CRP, C-reactive protein; DPI, diphenylene iodonium; IQR, interquartile range; HBD, healthy blood donors; MPO, myeloperoxidase; NETs, neutrophil extracellular traps; PMA, phorbol myristate acetate; *, p<0.05.)
