# Supplementary material for: Mapping autoantibody targets of full-length C-reactive protein in systemic lupus erythematosus: importance for neutrophil function and classical complement activation
Source: Front Immunol. 2025 May 15;16:1578372. doi: 10.3389/fimmu.2025.1578372 (PMC12119686; doi:10.3389/fimmu.2025.1578372)
Supplement: Supplementary file 3 [file Table1.docx]

Supplementary Table 1

Bovine Collagen Gel

**Table S1.** Bovine Collagen I gel (2mg/ml) composition.

|  | Amount (µl) | Comments | Manufacturer |
| --- | --- | --- | --- |
| Ingredients |  |  |  |
| 10x RPMI | 20 |  | Sigma-Aldrich, Saint Louis, MO, USA |
| NaOH (1M) | 15 |  |  |
| H_2_O | 10 |  |  |
| NaHCO_3_ (7.5%) | 5 |  | Thermo Fisher Scientific, Waltham, MA, USA |
| 1x RPMI | 50 | 2% fetal bovine serum, 2mM Glutamin (Sigma-Aldrich) | Sigma-Aldrich |
| Collagen I, Bovine (4mg/ml) | 150 | Diluted in 0.1 mM acetic acid | ibidi GmbH, Gräfelfing, Germany |
| Cell suspension (in 1x RPMI) | 50 |  |  |
